# Cognitive Task Performance During Titration Predicts Deep Brain Stimulation Treatment Efficacy: Evidence From a Case Study

**DOI:** 10.3389/fpsyt.2020.00030

**Published:** 2020-02-19

**Authors:** Emily R. Weichart, Per B. Sederberg, Francesco Sammartino, Vibhor Krishna, John D. Corrigan, Ali R. Rezai

**Affiliations:** ^1^Department of Psychology, Ohio State University, Columbus, OH, United States; ^2^Department of Neurosurgery, Ohio State University Wexner Medical Center, Columbus, OH, United States; ^3^Department of Physical Medicine and Rehabilitation, Ohio State University Wexner Medical Center, Columbus, OH, United States

**Keywords:** deep brain stimulation (DBS), cognitive testing, inhibitory control abilities, nucleus accumbens, morbid obesity, electroencephalography (EEG), diffusion tensor imaging (DTI)

## Abstract

Device titration is a major challenge when using deep brain stimulation (DBS) to treat behavioral disorders. Unlike in movement disorders, there is no reliable real-time clinical feedback for changes in complex behaviors resulting from DBS. Here, a female patient receiving DBS of the nucleus accumbens for the treatment of morbid obesity underwent cognitive testing *via* the flanker task alongside traditional methods of device titration. One set of stimulation parameters administered during titration resulted in acute cognitive improvement (p = 0.033) and increased frontal engagement as measured by electroencephalography (left anterior: p = 0.007, right anterior: p = 0.005) relative to DBS-OFF. The same parameters resulted in the most weight-loss during long-term continuous stimulation (47.8 lbs lost in 129 days) compared to the results of other stimulation settings. Diffusion tensor imaging analyses showed increased connectivity to dorsal attention networks and decreased connectivity to the default mode network for optimal parameters (p < 0.01). Our results provide evidence that targeted cognitive testing is a potentially useful tool for capturing acute effects of DBS stimulation during titration and predicting long-term treatment outcomes.

**Clinical Trial Registration:**

www.ClinicalTrials.gov, identifier: NCT01512134.

## Introduction

In the context of DBS, ‘titration’ is the process of adjusting stimulation parameters to reduce symptoms and avoid side effects. For movement disorders, DBS titration is typically accomplished through trial-and-error methods whereby clinicians sample combinations of device settings (i.e., electrode polarity, amplitude, pulse width, and frequency) and assesses acute clinical effects (1, 2). Trial-and-error methods have been successful when there is immediate, observable feedback (e.g., alleviation of Parkinson’s tremor) following device adjustment. When treating disorders of behavioral rather than movement regulation, however, trial-and-error methods become problematic. In contrast to the physical symptoms associated with movement disorders, behavioral disorders often do not include symptoms that can be objectively observed and measured in the clinical setting. The effects of stimulation can often take weeks or months to manifest (3), and it can take up to 1–2 years to determine the therapeutic window for stimulation settings [e.g., (4)].

There is an urgent need for a method of DBS titration that 1) relies on immediate effects with a latency of few minutes rather than weeks or months, 2) is objective, valid, and reliable, 3) is sensitive to incremental stimulation adjustments, 4) can be administered multiple times within a session without response biases, and importantly, 5) predicts long-term clinical results. Here, we propose a cognitive task-based method for acute stimulation assessment during nucleus accumbens (NAcc) DBS titration. In light of compelling evidence that cognitive performance is sensitive to stimulation loci and strengths (5), we investigated the possibility of using objective cognitive measures to guide the selection of optimal stimulation settings for NAcc DBS. Specifically, we chose an inhibitory control task to capture cognitive changes associated with different sets of stimulation parameters. Inhibitory control is broadly defined as the ability to suppress information that interferes with goal-driven behavior (6). Several lines of evidence have demonstrated that the NAcc plays a critical role in the complex mechanisms underlying inhibitory control, including lesion studies in rats and local field potential studies in humans (7).

In the current study, we measured one female participant’s ability to engage inhibitory control *via* the flanker task (8) while she independently underwent standard device titration procedures for DBS of the NAcc. We selected the flanker task for the current project in light of behavioral and electrophysiological evidence that obese participants have slower reaction times and reduced error-related negativity electroencephalography (EEG) activity during inhibitory control tasks compared to healthy controls (9–12). Additionally, functional magnetic resonance imaging (fMRI) work has shown that obese participants have reduced inhibitory control activity in the dorsolateral preforntal cortex [dlPFC; (13)] and anterior cingulate cortex [ACC; (14)]. These two regions have been exhaustively studied using the flanker task, with results demonstrating a direct relationship between dlPFC-ACC coactivation and flanker task performance (15–18).

Data collected during titration was analyzed retrospectively, after the patient had been identified as a DBS responder. Our primary hypothesis was that optimal device settings for long-term clinical outcomes would be linked to acute improvement in task performance during titration. This would support the idea that cognitive testing is a viable alternative to traditional methods of device titration and is a worthwhile avenue for investigation in future work with a larger cohort of patients. After identifying clinically effective settings, we performed diffusion tensor imaging (DTI) connectivity and EEG analyses to gain additional insight into the mechanisms underlying the observed stimulation effects. Given that DBS of the NAcc has been successfully implemented as a treatment for other behavioral disorders by regulating the frontal-thalamic pathway (19, 20), we hypothesized that optimal device settings would result in increased connectivity to frontal networks as measured by DTI as well as increased frontal engagement during the flanker task as measured by EEG. This study serves as a first step toward developing a task-guided tool for DBS titration, which has the potential to drastically improve the quality and efficiency of standard procedures for treating behavioral disorders.

## Materials and Methods

### Participants

One female participant completed this non-randomized phase I safety and feasibility prospective open label interventional pilot study investigating DBS as a treatment for obesity. Two other participants enrolled but did not complete the study (21) and did not reach responder criteria prior to the time of withdrawal. All participants met or exceeded the 40 kg/m^2^ body mass index classification threshold for morbid obesity, were at least 24 months post Roux-en-Y gastric bypass surgery without evidence of sustained weight loss, and were free of neurological or other severe medical conditions. Participants were recruited upon referral from a nutritionist. MRI scans at baseline confirmed that participants had no damage to the NAcc target in either hemisphere. The study met institutional requirements for research involving human subjects, was approved by the Food and Drug Administration (FDA) and Ohio State University’s Biomedical Sciences Human Subject Institutional Review Board (Protocol: 2011H0329), and was registered on ClinicalTrials.gov (Identifier: NCT01512134). All participants provided written informed consent after a full explanation of study procedures was provided by a clinician. The FDA approved an *a priori* responder criterion for minimal clinical effectiveness of 15% excess body weight loss. Details provided in the sections to follow refer only to the participant who completed the full study.

### Surgery

DBS leads (Medtronic Neurological Model 3391) were implanted bilaterally in the NAcc *via* frame-based stereotactic procedures. The anatomical target was visualized using standard axial, coronal, and sagittal T1, T2 and inversion-recovery MRI-guided methods. The Medtronic Stealth navigation system (Stealth Framelink software; Medtronic Inc., Minneapolis, MN) was used to simulate the planned lead trajectory and confirm avoidance of vasculature. Ventral contact locations relative to the midcommisural point were as follows: left hemisphere X = –6.51 mm, Y = 15.51 mm, Z = –5.22 mm; right hemisphere X = 7.36 mm, Y = 13.60 mm, Z = –5.36 mm, where X is medial-lateral, Y is anterior-posterior, and Z is rostral-caudal. Surgical coordinates for all contacts are provided in **Supplementary Table 1**. Lead locations within the NAcc were verified during surgery using single-cell microelectrode recordings and were subsequently confirmed using a fusion of pre-op T1 anatomical magnetic resonance imaging (MRI; 3T) and 1-month post-op computerized tomography (CT) scans. Anatomical reconstructions of lead placements were confirmed by an expert neuroanatomist. Reconstructed CT/MRI images of the implanted leads are shown in **Supplementary Figure 1**.

### Titration

Following a 6-week post-surgery recovery phase, the participant attended weekly 1 to 2-hour study visits with a physician for 12 weeks. During these study visits, device settings including polarity, amplitude, pulse width, and frequency were titrated based on the participant’s ratings of mood, energy, and anxiety, and avoidance of adverse side effects, per standard procedures. The physician used a systematic, iterative method to sample the parameter space of device settings during each visit. Contact stimulation could be monopolar or bipolar, and up to two contacts were stimulated in each hemisphere at once. Exploratory settings were applied for 5–15 minutes each.

During this procedure, the participant was intermittently asked to complete 1–5 blocks of the flanker task. The participant indicated the direction of a central arrow while ignoring congruent (e.g., <<<<<<<), incongruent (e.g., >>><>>>), or neutral (e.g., ooo<ooo) distractors. In the incongruent condition specifically, participants in the flanker task need to engage inhibitory control mechanisms to overcome influence of the distractor arrows and correctly identify the direction of the target. Each block contained 36 trials, 12 from each condition. A custom program using the State Machine Interface Library for Experiments (SMILE; https://github.com/compmem/smile) generated the randomized task lists, presented stimuli, and logged responses. Stimuli were presented on a standard 14-inch laptop screen. The participant pressed the ‘J’ and ‘K’ keys on the keyboard to indicate left and right target directions respectively. Stimuli remained on the screen until a response was made, and a fixation cross appeared for a jittered duration of 0.5–1 s in between stimuli. Cognitive testing *via* the flanker task was completely independent of device titration, such that the physician was not able to use the participant’s task performance to gauge the effectiveness of any particular device settings. Instead, the clinical research team made decisions about changing device settings based on week-to-week weight changes and the participant’s self-reports of mood and behavior.

### Long-Term Monitoring

After the titration phase, stimulation was continuous for the first 10 months of the 30-month long-term follow-up phase. In an effort to conserve the battery life of the device, we introduced a brief trial period of “cycling” stimulation during which the device was turned on during the day and automatically turned off at night. During this trial, we noted substantially-reduced battery consumption without negative reports from the patient, nor adverse effects on weight loss. For the latter 20 months of long-term follow-up, we therefore set the device to cycle between a 16- to 17-hour ‘ON’ state during the day and a 7- to 8-hour ‘OFF’ state at night. The participant attended monthly study visits for weight and body fat percentage measurements, nutritional counseling, and adverse effects monitoring. Stimulation parameters were adjusted as needed, based on participant feedback and in an effort to improve clinical effects. As in the titration phase, the flanker task was administered throughout long-term follow-up study visits if changes to the device settings were made. Weight and active stimulation settings were recorded each time a change was made, or at least once per month when settings were stable. Given that substantially more unique stimulation settings were tested during titration compared to long-term monitoring, stimulation settings were organized into bins based on active contacts and “high” or “low” voltage relative to 5V for the purposes of our analyses. The three sets of stimulation parameters that were represented in both the titration phase and the long-term monitoring phase were as follows: 1) bilateral lower middle contacts, low amplitudes; 2) bilateral lower middle contacts, high amplitudes; and 3) both bilateral middle contacts, high amplitudes.

### EEG Recording

The participant completed one EEG session after long-term stimulation settings had been in a clinically effective range for 14 days (treatment settings at the time of the session: LEFT: Case+ 1-, 5 V, 120 μs, 130 Hz; RIGHT: Case+ 9-, 3.5 V, 120 μs, 130 Hz). The participant completed two blocks of the flanker task (blocks 1–2) in a DBS-ON state. Bilateral stimulation was turned off using a Medtronic wireless DBS Patient Programmer, and the participant completed two blocks of the task in the DBS-OFF state (blocks 3–4). Stimulation was turned on again, and the participant completed two more blocks of the flanker task in the DBS-ON state (blocks 5–6). In order to capture acute effects of DBS, no more than two minutes passed from the time of switching the device ON or OFF to when a task block began. Blocks 1 and 2 were excluded from analyses in an effort to ensure that our results would be driven by the effects of stimulation rather than practice effects. Here, “practice effects” refer to incidental improvements in performance as participants acclimate to a task (22, 23).

Stimuli were presented and responses were recorded *via* a desktop PC connected to a 24” LCD display. The participant was fitted with an elastic cap embedded with 64 Ag-AgCl scalp electrodes arranged in an extended 10–20 array (BrainProducts GmbH, Munich, Germany) and seated in an electrically-shielded testing room. Electrodes were referenced to Cz. Signal was sampled at a rate of 1000 Hz *via* a DC-powered actiCHamp amplifier connected to a desktop PC. Electrode impedances were reduced to less than 25 Kohms prior to experimental testing, as recommended by the equipment manufacturer. EEG signal was monitored throughout the session for abnormalities using PyCorder software (BrainProducts GmbH, Munich, Germany) on the acquisition PC.

All EEG preprocessing was completed using custom functions in Python Time Series Analysis (PTSA; https://github.com/compmem/ptsa). Data were filtered from 0.25–20 Hz to eliminate low-frequency noise and electrical artifacts from the DBS generator. Wavelet-enhanced independent component analysis (24) removed artifacts from eye-blinks and saccades.

### Connectivity

Patterns of structural connectivity were assessed using pre-op diffusion-weighted imaging (60 diffusion directions, 2 mm iso-voxel, Philips Ingenia CX). The structural (T1-weighted and post-operative CT images) and diffusion-weighted images were co-registered and nonlinearly aligned to the MNI152 T1 template space. Despite only analyzing data from one subject, we co-registered images to MNI space in order to overcome the limitations of morphing standard network atlases to a single brain, as well as provide results in an interpretable, universal space. The volumes of tissue activation were calculated for each stimulation parameter set and electrode impedance using methods described by [(25); also in (26)]. The volumes of tissue activation for the same three sets of stimulation parameters used in our behavioral analyses were used as seeds for probabilistic tractography (FSL, three fibers per voxel model, 25000 samples per voxel). A validated cortical multimodality atlas was used to generate target masks (27). The connectivity maps resulting from each individual voltage change were thresholded to the 99th percentile of ‘robust’ intensity and were subjected to a linear mixed-effects model using AFNI’s 3dMVM program (28) with the amplitude of stimulation as the within-subject variable. The resulting statistical maps thresholded by the false discovery rate corrected p-value of <0.01 were clusterized using an unsupervised density-based clustering algorithm [DBSCAN-R library; (29)] with parameters epsilon=1.6 and minimum points=5. The measure of connectivity and directionality (i.e, increased vs. decreased connectivity) was interpreted based on the number of voxels intersecting the network masks, following a methodology reported by (30).

## Results

### Optimal Stimulation Settings for Weight Loss

**Figure 1:**
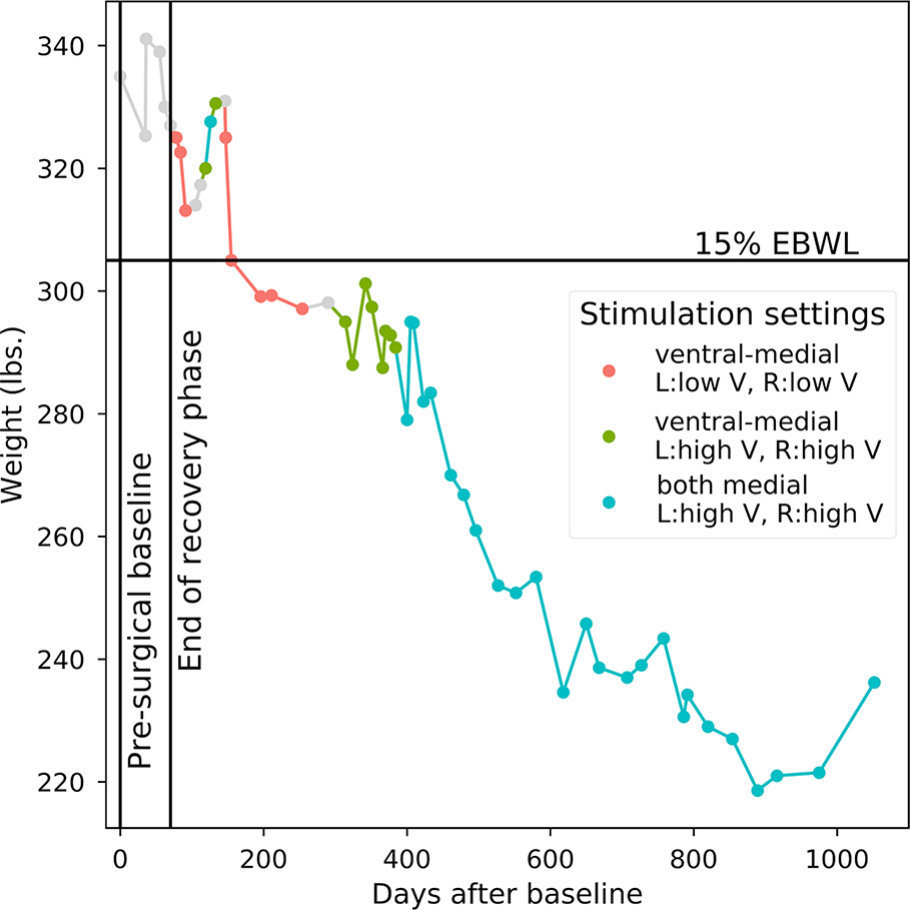
Weight progression. Starting point is 335 lbs. at the pre-surgical baseline. Points correspond to individual weight measurements. Line colors correspond to the long-term device settings that were active in the period of time prior to each weight measurement. OTHER: Points 1–6 include pre-surgical baseline and post-surgery recovery when no stimulation occurred; stimulation parameters could not be verified between points 14 and 15 and between points 20 and 21; insufficient titration and long-term data for evaluating active stimulation parameters between points 9 and 11.

Approximately 5 months after the device was turned on, the participant reached *a priori* responder criteria of 15% excess body weight loss. Over the course of the 36-month study, the participant lost a total of 98.8 pounds equal to 47% of her excess body weight, dropping from an initial body mass index of 55.8 to 39.3. Three sets of stimulation parameters were tested for long-term effects: (1) Bilateral stimulation of ventral-medial contacts at amplitudes less than or equal to 5V (minimum of 2V) resulted in the fastest rate of weight loss (47.80 lbs lost over the course of 129 days of stimulation, mean change of –0.37 lbs/day). We therefore determined *post hoc* that these were the optimal settings for weight loss in this participant. (2) Stimulating the same contacts at higher amplitudes (>5V, maximum of 9V) was associated with minimal weight loss (1.61 lbs lost over the course of 108 days of stimulation, mean change of –0.01 lbs/day). (3) Both bilateral medial contacts were stimulated at high amplitudes (>5V, maximum of 8V) for most of the study’s duration, resulting in a substantial net weight loss (47.00 lbs lost over the course of 675 days), but at a less striking rate than optimal (mean change of –0.07 lbs/day). **Figure 1** shows the participant’s weight progression throughout the study.

### Task Performance

Overall accuracy across all task blocks was near ceiling (accuracy across all conditions: 0.988, accuracy for incongruent trials only: 0.982). Task data from all visits were sorted based on active contacts and stimulation amplitudes relative to 5V. To remove within-session practice effects, the first block from each visit was excluded from further analyses (22, 23). Since the incongruent task condition is most relevant for measuring inhibitory control, we only considered incongruent trials in our analysis.

Incorrect trials and reaction time (RT) outliers were removed. RTs and within-trial trial numbers were log-transformed in an effort to satisfy normality assumptions (31, 32). Trial numbers since stimulation change were log-transformed. Data from each stimulation parameter set were individually compared to DBS-OFF (33). Log RTs were analyzed using likelihood ratio tests of mixed-effects models where the factors were DBS status (ON, OFF) and log trial number. By-run intercepts and random slopes for the interaction terms were included as random effects.

**Figure 2:**
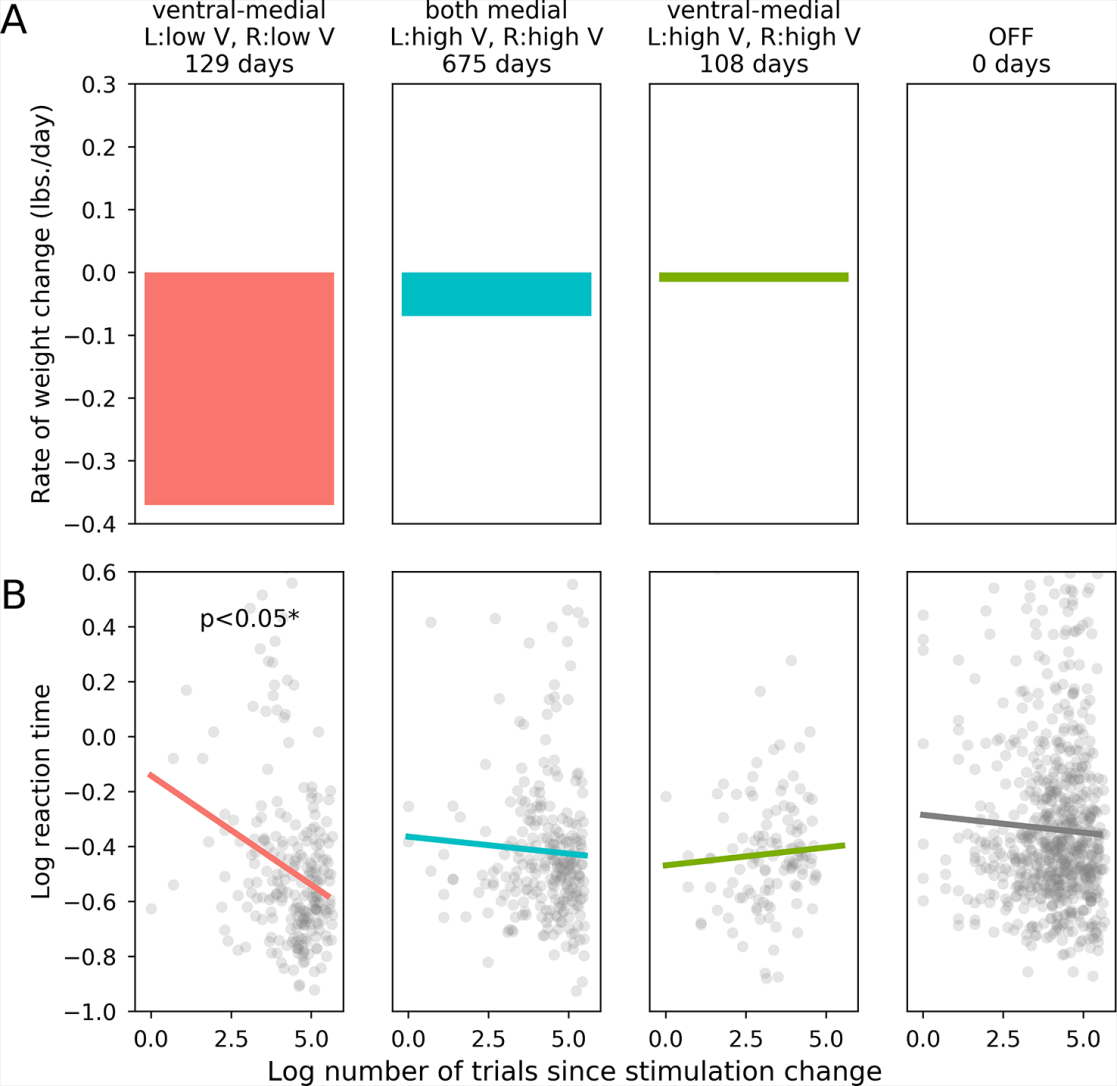
Effects of stimulation on weight loss and flanker performance. **(A)**: Rate of long term weight loss (mean lbs. per day) for each set of stimulation parameters. **(B)**: The horizontal axis is the log-transformed trial number, which is an indicator of how much time had passed since the relevant stimulation parameter set became active. The vertical axis is the patient’s log-transformed reaction time on each trial, which was our dependent variable metric for the patient’s performance on the flanker task.

Following activation of optimal stimulation parameters (as determined by mean rate of weight loss; bilateral ventral-medial contacts, low amplitudes), the participant made significantly faster correct responses to incongruent task items compared with the device that was turned OFF [*X*^2^(1) = 4.571, p = 0.033]. The participant’s RTs were not significantly affected by stimulation with any other parameter sets sampled during titration, including those tested for long-term treatment effects (bilateral ventral-medial contracts, high amplitudes: *X*^2^(1) = 0.301, p = 0.580; both bilateral medial contacts, high amplitudes: *X*^2^(1) = 0.255, p = 0.613). Long-term rate of weight loss and acute cognitive performance (as measured by log trial-level RTs in the incongruent task condition) for each set of stimulation settings are shown in **Figure 2**. Direct comparisons between optimal and sub-optimal parameter sets did not yield statistically significant results and are reported in the Supplementary Materials. Comparing each active stimulation condition to DBS-OFF for correct RTs in the congruent condition also did not yield statistically significant results (bilateral ventral-medial contacts, low amplitudes: *X*^2^(1) = 1.038, p = 0.308; both bilateral medial contacts, high amplitudes: *X*^2^(1) = 2.562, p = 0.110; bilateral ventral-medial contacts, high amplitudes: *X*^2^(1) = 0.0045, p = 0.947). Since the corresponding comparisons for the incongruent task condition produced a statistically significant result for optimal stimulation parameters, this supports the idea that our effects are specifically related to inhibitory control rather than simply being an RT effect.

### EEG

Incongruent trials were segmented into events and time-locked to stimulus onset. Events were 3000 ms long, beginning 1500 ms pre-stimulus onset and baseline-adjusted to 100 ms pre-stimulus. Events were rejected if kurtosis exceeded 5.0 or if amplitude range exceeded 100 V. Electrodes were grouped into 4 quadrants, representing left anterior, right anterior, left posterior, and right posterior regions (left anterior: F1, F3, FC1, FC3, FC5, C1, C3, C5; right anterior: F2, F4, FC2, FC4, FC6, C2, C4, C6; left posterior: CP1, CP3, CP5, P1, P3, P5, PO3, PO7; right posterior: CP2, CP4, CP6, P2, P4, P6, PO4, PO8).

The participant performed with high accuracy throughout the EEG session (accuracy across all conditions: 0.985, accuracy for incongruent trials only: 0.986). RTs slowed after DBS was turned from ON to OFF (slope = 0.067) and became faster when DBS was turned from OFF to ON (slope = –0.021). These results, however, were not statistically significant (ON to OFF: R^2^ = 0.164, p = 0.443, α = 0.05; OFF to ON: R^2^ = –0.037, p = 0.864, α = 0.05).

**Figure 3:**
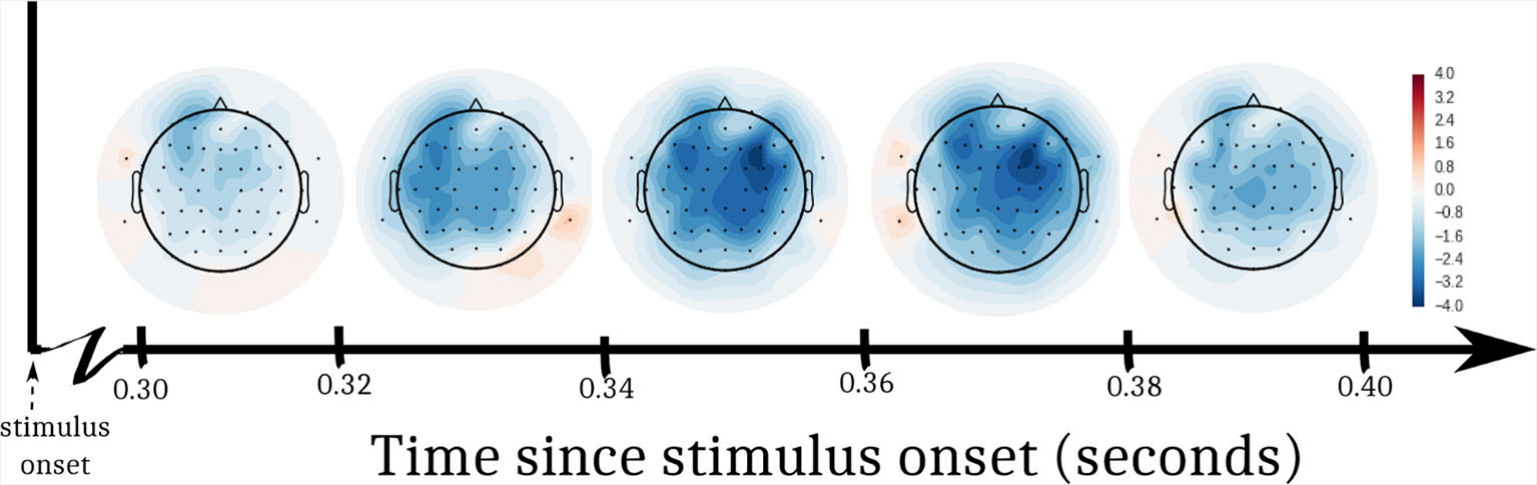
T-statistics from EEG analysis. We performed an ANOVA at each electrode to predict EEG voltage. Log trial number and DBS status (ON, OFF) were factors. The interaction of the factors was a significant predictor of EEG voltage in the left and right frontal quadrants of the participant’s scalp. Swaths of color represent t-values from the ANOVA within 5 sub-windows of time after the stimulus appeared.

A 2-way ANOVA predicted EEG amplitude from the interaction of DBS status (ON, OFF) and log trial number following a change in stimulation. In order to assess EEG activity during the decision interval within each trial, we defined a post-stimulus time window of interest from 300–400 ms. This window has been selected for assessing voltage differences between congruent and incongruent flanker stimuli in past studies (34), and it allowed us to ignore irrelevant artifacts from early perceptual processes and motor-planning. **Figure 3** shows t-values from the ANOVA at each electrode, split into 5 equal time bins within the window of interest. In both the left and right anterior quadrants, there was a drop in amplitude through time after DBS was turned OFF compared to when it was turned ON. Correcting for multiple comparisons, these effects were significant in anterior quadrants [left anterior: F(28,27) = 8.52, p = 0.007, α = 0.0125; right anterior: F(28,27) = 9.23, p = 0.005, α = 0.0125]. We did not observe a significant change in amplitude in posterior quadrants [left posterior: F(28,27) = 4.73, p = 0.038, α = 0.0125; right posterior: F(28,27) = 4.33, p = 0.047, α = 0.0125].

### Connectivity

**Figure 4:**
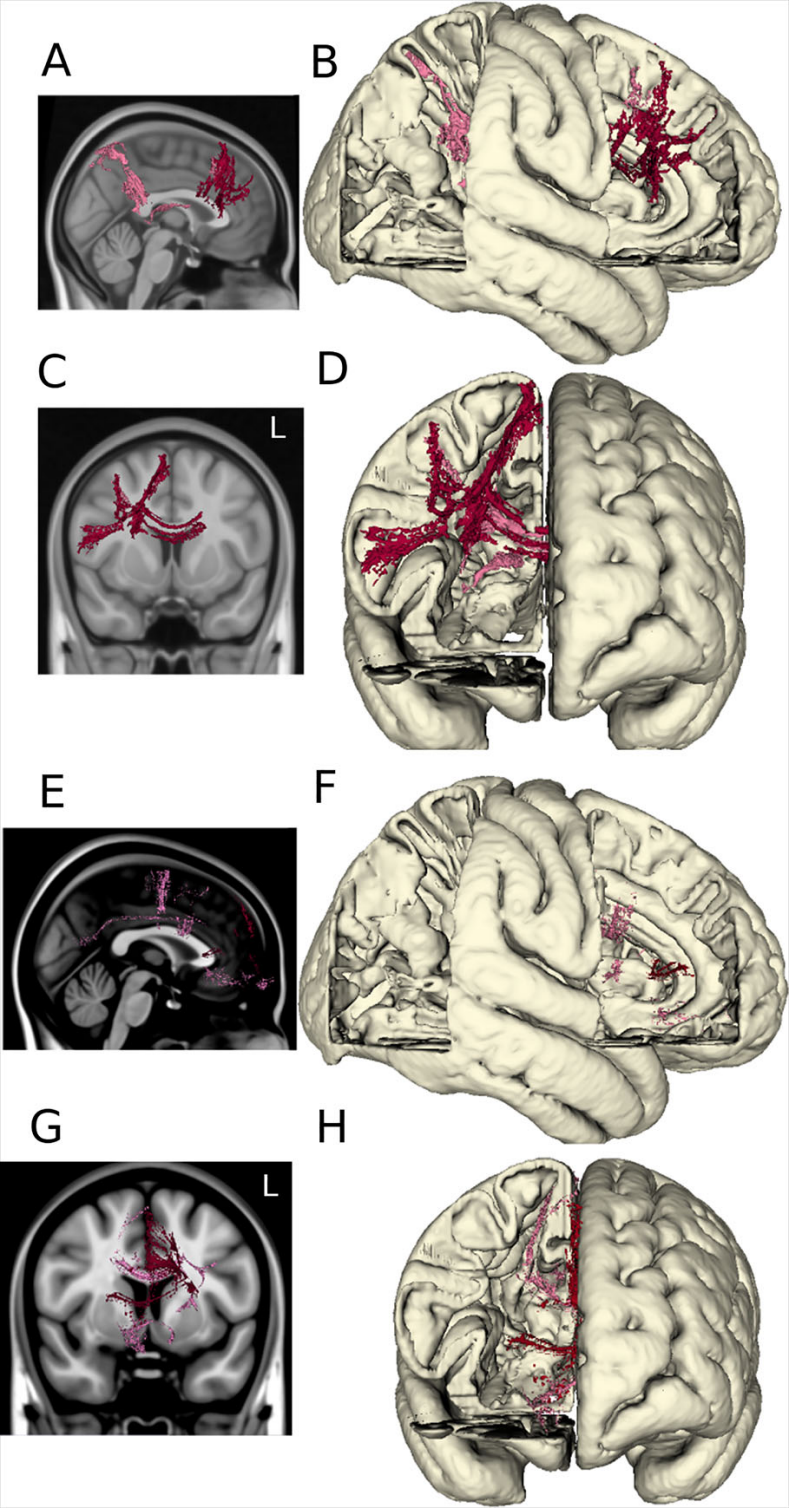
DTI tractography. The probabilistic connectivity maps at optimal and optimal vs. suboptimal DBS settings are shown in sagittal and coronal projections with their respective 3D models. *Panels* **(A**–**D)**: Significant voxels associated with optimal DBS settings (bilateral lower middle contacts, low amplitudes). *Panels* **(E**–**H)**: Significant voxels comparing optimal vs. suboptimal DBS settings (bilateral lower middle contacts, high amplitudes).

Volumes of tissue activation associated with the three stimulation settings of interest were used as seeds for probabilistic tractography with target masks derived from a multimodality cortical atlas. The probability of connectivity was determined based on the number of voxels intersected by tractography in each network mask for each of the three settings. Optimal DBS settings were associated with higher probability of connectivity in the right dorsolateral and dorsomedial prefrontal cortex as shown in **Figures 4A–D**. When comparing the optimal versus suboptimal settings, the significant cortical clusters were localized within frontal regions (basal frontal, cingulate) as shown in **Figures 4E–H**. The MNI coordinates of the largest clusters are summarized in **Supplementary Tables 2–6**.

## Discussion

### Summary

Standard trial-and-error methods of DBS device titration depend on immediate, measurable effects of individual sets of stimulation parameters. As clinical applications for DBS have expanded beyond movement disorders, device titration methods have not been adequately adapted for behavioral disorders lacking overt physical symptoms. While current methods rely on subjective ratings of mood, energy, and anxiety to guide the selection of parameters for long-term stimulation, we investigated cognitive task performance as a possible alternative. Based on previous work that has defined the role of the NAcc within a complex cognitive architecture (35), we hypothesized that acute performance on an inhibitory control task during device titration could predict long-term treatment efficacy. Converging evidence from the current preliminary study indeed suggested a link between acute cognitive performance and subsequent clinical outcomes as determined by retrospective analyses. Given this preliminary evidence, the next step will be to conduct a larger study where we can formally compare outcomes for groups of patients under standard versus task-guided device titration protocols.

### Interpretation of Results

A participant receiving NAcc DBS for the treatment of obesity completed blocks of the flanker task alongside traditional methods of device titration. Post-hoc linear mixed effects regression analyses indicated that the DBS settings linked to the fastest rate of weight loss produced an immediate, significant improvement in task performance. This finding is in line with previous work investigating acute changes in task performance related to different DBS-ON states as a way to tangentially assess stimulation efficacy. Mikos and colleagues (36), for example, used a computational model-based approach to link volumes of tissue activation at different contacts during DBS of the subthalamic nucleus to letter fluency in Parkinson’s disease patients. Their results suggested that cognitive performance correlates with treatment effects in motor disorders. Our results show that this connection potentially holds for behavioral disorders as well, even in cases when treatment effects are not immediately observable.

EEG results provided further insight into the neural mechanisms underlying the optimal DBS settings. DBS within the optimal parameter range resulted in a significant difference in cortical amplitude at frontal electrodes compared to when DBS was OFF. These are the results that we would expect, given that cognitively normal subjects show a higher-amplitude peak in frontocentral electrodes in EEG during inhibitory tasks (37). We believe our EEG results reflect higher engagement of conflict monitoring processes when optimal DBS settings are active. Further work will need to determine how EEG effects are linked to long-term treatment outcomes, but these results are nevertheless in line with positron emission tomography and fMRI evidence of frontal dysfunctions in obese participants. In particular, obese individuals have reduced activity related to inhibitory control in the dlPFC (13) and ACC (14). As indicated by our results, DBS of the NAcc may be modulating these frontal networks and thus counteracting this hypoactivity and associated lack of inhibitory control in our participant.

While low amplitude stimulation at ventral-medial contacts was optimal for weight loss, increasing amplitudes above 5V at the same contacts both diminished cognitive task performance and caused the participant’s weight loss to slow. Whereas high-amplitude stimulation is often used to achieve treatment effects by mimicking tissue lesions (38, 39), this is not necessarily a desirable approach for all cases. Our DTI connectivity analyses illustrate why low amplitude stimulation proved to be effective for treatment in this case while high amplitude stimulation did not. Low amplitude stimulation significantly increased connectivity to dorsal attention networks and simultaneously decreased connectivity to the default mode network. High amplitude stimulation, on the other hand, resulted in expansive, nonspecific connectivity without a significant advantage of any network in particular. High-amplitude NAcc DBS has been argued to benefit obsessive compulsive disorder due to blockade effects within an otherwise hyperactive information processing network connecting the basal ganglia, amygdala, thalamus, and prefrontal cortex (20). For a disorder like obesity that is characterized by a *hypo*active frontal-thalamic pathway (19), however, an approach geared toward targeted upregulation rather than attenuation appears to be more appropriate. Weight-loss alongside low-amplitude DBS of the NAcc was also recently observed in a handful of case studies [(40–42) see (43) for recent review].

In order for cognitive testing to be a viable tool for titration, it is important to choose a cognitive task that is relevant to both the stimulation target and the behavioral disorder of interest. Selecting the flanker task for the present study involved careful consideration of NAcc function and its relationship to obesity. Critically located in the basal forebrain, the NAcc serves as a hub of communication among limbic (ventral tegmental area, substantia nigra, and basolateral amygdala), motor (pallidum and striatum), and executive functioning (prefrontal cortex) networks. Given its proximal and functional relationships with several key structures in the reward pathway, NAcc stimulation has been proposed to modulate mood, impulsivity, and reward-seeking behaviors *via* dopaminergic signaling (44, 45). The role of the NAcc in inhibitory control was of particular interest in the present study, with compelling support from animal literature showing that NAcc stimulation affects inhibitory control on an immediate time scale (35). Furthermore, evidence from local field potential recordings in humans showed that inhibitory control paradigms such as the flanker task specifically engage the NAcc (46, 47). Our study aimed to capitalize on the relationship between the NAcc, inhibitory control, and obesity to link immediate effects of DBS to treatment efficacy.

### Conclusions

We propose that task-based titration can be extended beyond the flanker task and the NAcc, and future work will further investigate how we can use acute cognitive performance to predict long-term treatment outcomes. Though the implications of our results are limited due to our sample size, we have provided preliminary evidence that cognitive testing may be a valuable tool for titration. The next step will be to conduct a formal investigation with more participants and to compare clinical outcomes for groups being treated under standard versus task-guided device titration protocols.

## Data Availability Statement

The raw data supporting the conclusions of this article will be made available by the authors, without undue reservation, to any qualified researcher.

## Ethics Statement

The studies involving human participants were reviewed and approved by Ohio State University’s Biomedical Sciences Human Subject Institutional Review Board. The patients/participants provided their written informed consent to participate in this study.

## Author Contributions

AR designed the overall study with contributions from PS, VK, and JC. AR performed the surgery. AR and VK monitored the patient throughout the study. PS constructed the computerized cognitive task. EW collected and analyzed behavioral and EEG data with PS. FS and VK collected and analyzed DTI data. EW wrote the manuscript. AR, PS, VK, JC, and FS interpreted findings, discussed, and edited the manuscript.

## Funding

This research was supported by the Neurological Research Institute, the Ohio State University.

## Conflict of Interest

AR discloses holding equity positions and serving on the board of directors of the following organizations: Neurotechnology Innovation Translator (NIT) and Management (NIM), Sollis Therapeutics, and Autonomic Technologies (ATI). AR also discloses receiving payment as a consultant at ATI.

The remaining authors declare that the research was conducted in the absence of any commercial or financial relationships that could be construed as a potential conflict of interest.
