# Supplementary material for: Cognitive Task Performance During Titration Predicts Deep Brain Stimulation Treatment Efficacy: Evidence From a Case Study
Source: Front Psychiatry. 2020 Feb 19;11:30. doi: 10.3389/fpsyt.2020.00030 (PMC7043267; doi:10.3389/fpsyt.2020.00030)
Supplement: Supplementary file 1 [file DataSheet_1.docx]

***Supplementary Materials***

**Additional Results**

Mean and standard deviations of RTs within the stimulation parameter sets of interest across titration sessions were as follows:

1. Lower-middle contacts, low V (optimal): mean=0.582s, sd=0.182s
2. Both middle contacts, high V: mean=0.592s, sd=0.182s
3. Lower-middle contacts, high V: mean=0.599s, sd=0.151s
4. DBS-OFF: mean=0.633s, sd=0.182s

Results comparing correct RTs in the incongruent condition during stimulation with optimal stimulation parameter values (low V, ventral-medial contacts) to either set of suboptimal parameter values were not statistically significant:

1. Lower-middle contacts, low V (optimal) vs. both middle contacts, high V: chisq(1)=2.276, p=0.131
2. Lower-middle contacts, low V (optimal) vs. lower-middle contacts, high V: chisq(1)=1.164, p=0.281

Results comparing correct RTs in the congruent condition during stimulation with optimal parameter values to either set of suboptimal parameter values were also not significant:

1. Lower-middle contacts, low V (optimal) vs. both middle contacts, high V: chisq(1)=0.327, p=0.567
2. Lower-middle contacts, low V (optimal) vs. lower-middle contacts, high V: chisq(1)=0.167, p=0.682

Results comparing correct RTs in the neutral condition during stimulation for each parameter set to DBS-OFF were not significant:

1. Lower-middle contacts, low V (optimal) vs. DBS-OFF: chisq(1)=1.972, p=0.160
2. Both middle contacts, high V vs. DBS-OFF: chisq(1)=0.166, p=0.684
3. Lower-middle contacts, high V vs. DBS-OFF: chisq(1)=0.359, p=0.543

Long-term results were presented as rate of weight loss, calculated by dividing the total weight change by the total number of days that each set of stimulation parameters were active. As each set of parameters were active for very different amounts of time (129, 675, and 108 days), our rate calculations may have been biased by duration-dependent changes in stimulation efficacy. In a follow-up analysis, we therefore considered weight loss only within the initial period of active stimulation in an effort to standardize these long-term durations. Although durations were not exactly equal because we did not collect weight data on every day of the study, we find that the results presented in the main manuscript are maintained:

1. Lower-middle contacts, low V: 47.8 lbs lost in 129 days
2. Both middle contacts, high V: 22.2 lbs lost in 119 days
3. Lower-middle contacts, high V: 1.6 lbs lost in 108 days

For the main analyses, voltages were assigned to “low” and “high” groups relative to 5V. To demonstrate that our results are maintained beyond these specific group designations, we ran follow-up analyses with three voltage groups, each covering an equal range of 2.67V: low (2.00 to 4.67V), medium (4.67 to 7.33V), and high (7.33 to 10.00V). Using the same linear mixed effects regression analysis described in the manuscript, we identified a significant flanker RT effect for only one set of stimulation parameters: bilateral lower-middle contacts with medium V in the left hemisphere (L) and low V in the right hemisphere (R; chisq(1)=6.350, p=0.012). Applying these group delineations to the long-term weight loss data, we found that the same set of parameters that yielded a significant acute task effect also yielded the fastest rate of weight loss in the long term:

1. Lower-middle contacts, L medium V, R low V: 45.8 lbs lost in 121 days

Another set of parameters yielded the same amount of weight loss, but over a much longer period of time:

1. Both middle contacts, L high V, R high V: 45.8 lbs lost in 611 days

Other results were as follows:

1. Lower-middle contacts, L low V, R low V: 2.0 lbs lost in 8 days
2. Lower-middle contacts, L medium V, R medium V: 5.7 lbs gained in 14 days
3. Lower-middle contacts, L high V, R high V: 7.3 lbs lost in 94 days
4. Both middle contacts, L medium V, R medium V: 1.2 lbs lost in 64 days

**Supplementary Figure 1: Orientation of implanted leads.** Postoperative anterior-posterior (A) and lateral (B) x-ray images. Fusions of coronal (C) and left hemisphere sagittal (D) pre-op T1 anatomical MRI (3T) and 1-month post-op CT images showing lead placement.

*
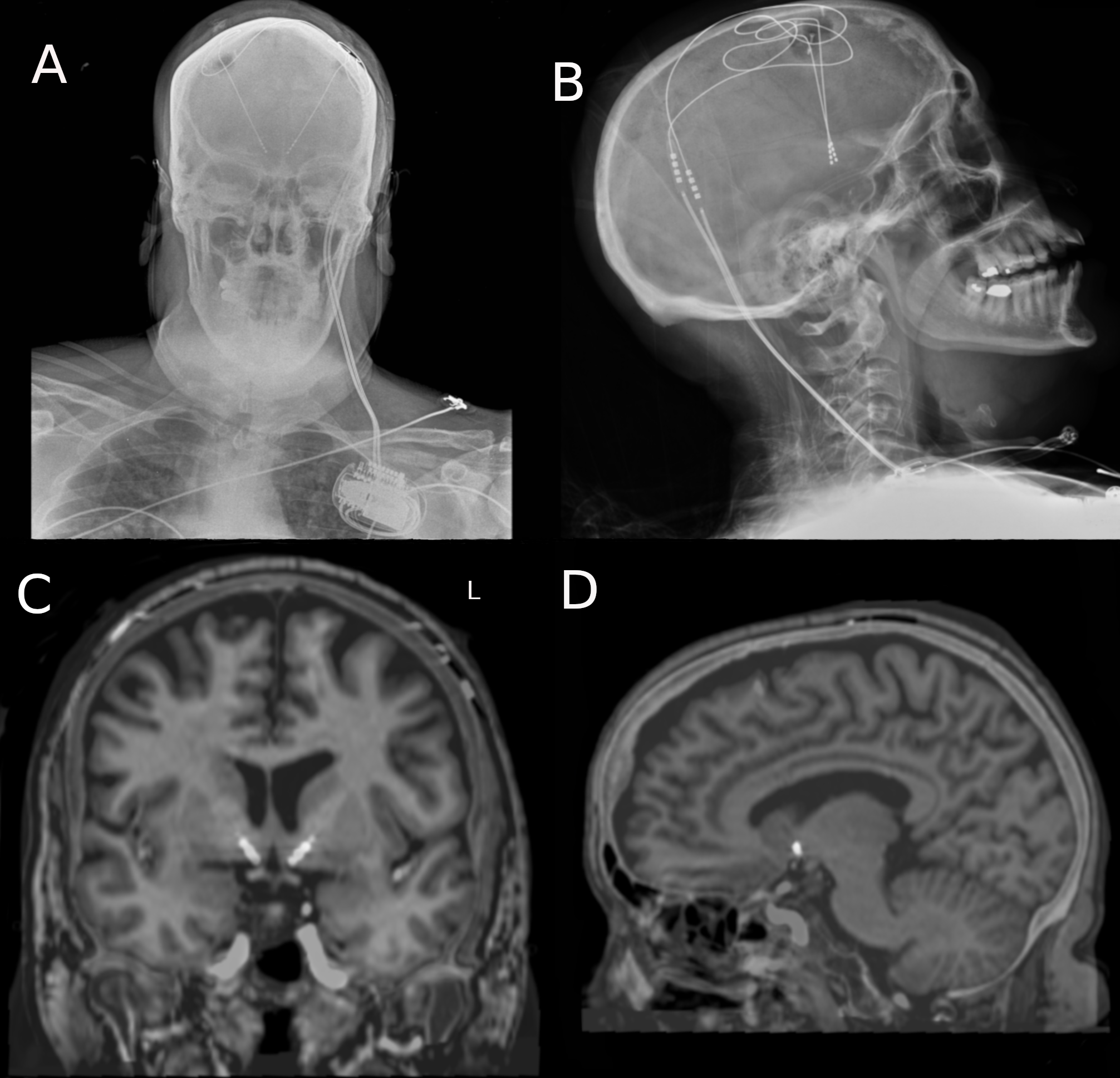
*

**Supplementary Table 1:** Contact locations. All coordinates are reported relative to the midcommisural point (MCP). X=medial-lateral, Y=anterior-posterior, Z=rostral-caudal.

| **Contact** | **Left** | | | **Right** | | |
| --- | --- | --- | --- | --- | --- | --- |
|  | **X** | **Y** | **Z** | **X** | **Y** | **Z** |
| 0 | -6.51mm | 15.51mm | -5.22mm | 7.36mm | 13.60mm | -5.36mm |
| 1 | -7.70mm | 16.30mm | -2.52mm | 8.76mm | 14.79mm | -2.98mm |
| 2 | -8.87mm | 17.07mm | -0.13mm | 10.15mm | 15.97mm | -0.60mm |
| 3 | -10.04mm | 17.85mm | 2.78mm | 11.54mm | 17.15mm | 1.78mm |

**Supplementary Table 2:** Clusters associated with optimal settings (bilateral lower middle contacts, low amplitudes) with center of mass coordinates in MNI space.

| Cluster# | Voxel# | CMx | Cmy | CMz |
| --- | --- | --- | --- | --- |
| 31 | 13751 | 148 | 297 | 206 |
| 1 | 11428 | 218 | 143 | 70 |
| 34 | 10058 | 149 | 154 | 212 |
| 44 | 2573 | 203 | 145 | 244 |
| 28 | 1608 | 148 | 210 | 169 |
| 37 | 825 | 206 | 174 | 187 |
| 51 | 502 | 117 | 285 | 232 |
| 11 | 458 | 189 | 182 | 128 |

**Supplementary Table 3:** Clusters associated with suboptimal settings (bilateral lower middle contacts, high amplitudes ) with center of mass coordinates in MNI space.

| Cluster# | Voxel# | CMx | Cmy | CMz |
| --- | --- | --- | --- | --- |
| 5 | 29739 | 212 | 157 | 70 |
| 199 | 23211 | 149 | 284 | 201 |
| 215 | 7701 | 144 | 149 | 232 |
| 276 | 3792 | 157 | 240 | 257 |
| 106 | 3642 | 166 | 364 | 131 |
| 238 | 2657 | 196 | 345 | 221 |
| 44 | 1417 | 157 | 301 | 83 |
| 294 | 1118 | 203 | 143 | 250 |
| 112 | 846 | 185 | 187 | 136 |
| 102 | 818 | 220 | 233 | 145 |
| 256 | 813 | 184 | 233 | 201 |
| 85 | 753 | 152 | 248 | 120 |
| 189 | 720 | 227 | 274 | 160 |
| 197 | 700 | 140 | 200 | 172 |
| 303 | 677 | 236 | 274 | 240 |
| 223 | 607 | 205 | 171 | 185 |
| 48 | 598 | 197 | 290 | 82 |
| 233 | 503 | 232 | 322 | 194 |
| 51 | 492 | 186 | 115 | 91 |
| 128 | 440 | 189 | 211 | 129 |
| 307 | 429 | 149 | 220 | 252 |
| 4 | 426 | 200 | 164 | 67 |
| 113 | 409 | 207 | 242 | 126 |
| 45 | 378 | 225 | 91 | 87 |
| 153 | 350 | 164 | 316 | 139 |
| 83 | 339 | 198 | 255 | 112 |
| 295 | 317 | 125 | 283 | 224 |
| 288 | 284 | 165 | 225 | 213 |
| 171 | 279 | 143 | 242 | 151 |

**Supplementary Table 4:** Clusters associated with suboptimal settings (both bilateral middle contacts, high amplitudes) with center of mass coordinates in MNI space.

| Cluster# | Voxel# | CMx | Cmy | CMz |
| --- | --- | --- | --- | --- |
| 109 | 28303 | 158 | 310 | 189 |
| 151 | 25432 | 172 | 151 | 222 |
| 8 | 24806 | 218 | 152 | 71 |
| 61 | 1439 | 156 | 233 | 125 |
| 254 | 992 | 157 | 238 | 253 |
| 98 | 884 | 120 | 263 | 132 |
| 242 | 665 | 120 | 285 | 230 |
| 218 | 636 | 175 | 198 | 200 |
| 227 | 634 | 134 | 321 | 207 |
| 82 | 586 | 137 | 219 | 126 |
| 217 | 335 | 208 | 99 | 204 |
| 179 | 298 | 213 | 118 | 182 |
| 127 | 297 | 118 | 182 | 151 |
| 222 | 292 | 186 | 234 | 198 |
| 183 | 260 | 134 | 196 | 177 |

**Supplementary Table 5:** Clusters from the general linear t-test between stimulation parameters associated with ‘improvement’ and ‘no change’ with center of mass coordinates in MNI space.

| Cluster# | Voxel# | CMx | Cmy | CMz |
| --- | --- | --- | --- | --- |
| 85 | 7326 | 193 | 345 | 189 |
| 138 | 3973 | 183 | 270 | 198 |
| 68 | 1961 | 172 | 325 | 126 |
| 149 | 1660 | 157 | 238 | 259 |
| 1 | 1577 | 216 | 158 | 65 |
| 133 | 1180 | 164 | 170 | 199 |
| 63 | 636 | 172 | 388 | 114 |
| 134 | 546 | 233 | 319 | 192 |
| 113 | 519 | 226 | 273 | 159 |
| 156 | 482 | 160 | 284 | 250 |
| 141 | 359 | 166 | 278 | 210 |
| 140 | 342 | 181 | 233 | 202 |

**Supplementary Table 6**: Clusters from the general linear t-test between stimulation parameters associated with ‘improvement’ and ‘worsening’ with center of mass coordinates in MNI space.

| Cluster# | Voxel# | CMx | Cmy | CMz |
| --- | --- | --- | --- | --- |
| 63 | 5458 | 160 | 154 | 211 |
| 1 | 5410 | 221 | 154 | 70 |
| 70 | 428 | 171 | 271 | 195 |
| 86 | 417 | 153 | 300 | 233 |
| 57 | 363 | 95 | 294 | 188 |
| 110 | 334 | 193 | 139 | 265 |
